# Systemic Treatment for Adults with Synovial Sarcoma

**DOI:** 10.1007/s11864-018-0525-1

**Published:** 2018-03-07

**Authors:** Ingrid M. E. Desar, Emmy D. G. Fleuren, Winette T. A. van der Graaf

**Affiliations:** 1 Department of Medical Oncology, Radboud University Medical Centre Nijmegen, Nijmegen, The Netherlands; 2 The Institute of Cancer Research, Sutton, UK; 3 The Institute of Cancer Research and the Royal Marsden NHS Foundation Trust, London, UK

**Keywords:** Synovial sarcoma, Chemotherapy, Targeted therapy, Immunotherapy

## Abstract

Synovial sarcoma (SS) is a rare, yet highly malignant, type of soft tissue sarcoma (STS), for which survival has not improved significantly during the past years. In this review, we focus on systemic treatment in adults. Compared to other STS, SS are relatively chemosensitive. Ifosfamide and ifosfamide combinations are active in different lines of treatment. In high-risk extremity and chest wall STS, neoadjuvant doxorubicin and ifosfamide has shown as much activity as high-dose ifosfamide. There are indications that combination chemotherapy with doxorubicin and ifosfamide in this setting improves outcome. In the first-line metastatic setting, combination treatment with doxorubicin and ifosfamide is a preferred option in fit patients, while in other patients, sequential doxorubicin and ifosfamide can be considered. In second and later lines, pazopanib and trabectedin have shown activity. Many new approaches to treat metastatic SS are currently under investigation, both preclinical as well as clinical, including other receptor tyrosine kinase inhibitors, epigenetic modulators, compounds interfering with DNA damage response (DDR), and immunotherapy.

## Introduction

Soft tissue sarcomas (STS) are rare, malignant mesenchymal tumors that include over 70 different and highly diverse histological subtypes. Synovial sarcoma (SS) accounts for 5–10% of all STS [1, 2]. SS is an intriguing disease as, unlike the majority of STS, it can occur at any age and everywhere in the body. The peak incidence is in the 30s and it often presents in the extremities [3•]. The incidence of SS continued to increase from 0.906 to 1.548 per 1,000,000 between 1983 and 2012 [4]. Despite its name, SS does not arise from the synovium, neither expresses synovial markers [5]. SS was initially described as a biphasic neoplasm comprising of both epithelial and uniform spindle cell components. SS is characterized by the presence of the pathognomonic *t*(*X*;18) (p11.2;q11.2) translocation, involving a fusion of the *SS18* (formerly *SYT*) gene on chromosome 18 to one of the synovial sarcoma *X* (*SSX*) genes on chromosome X (usually *SSX1* or *SSX2*), which is seen in more than 90% of SS and results in the formation of *SS18-SSX* fusion oncogenes [6].

For localized non high-risk disease, treatment consists of surgery, on indication combined with (neo)adjuvant radiotherapy. In about 50% of cases, metastases occur [7]. Interestingly, the prognosis of primary non metastasized SS is related to the age of the patient, with a much better relative survival in children compared to older patients, and more genomic instability with increasing age [3•, 8]. The 5-year overall survival (OS) for all SS is 60.5%, but is largely age-dependent [4].

In most cases, metastases are localized in the lung (80%), although metastases can arise in lymph nodes (up to 20%), bone (9.9%), and liver (4.5%) as well [9•, 10]. Once metastasized, curative treatment is hardly achievable, with the exception of late and resectable oligometastatic disease and patients are treated with chemotherapy with a palliative intent. Compared to STS as a group, SS is more sensitive to certain chemotherapeutic agents [9•, 11].

For long, STS have been clinically treated as one type of disease, and most chemotherapy trials included the majority of STS subtypes. The first attempt to address the differences in tumor behavior led to stratification for leiomyosarcomas, liposarcomas, SS, and the so-called other group and made use of the 3- and 6-month progression-free rate (PFR) in second- and higher-line studies [12].

It is only in recent years that more sarcoma subtype-specific trials are undertaken, recognizing the large diversity in clinical behavior, biology, and genetic make-up of the different STS and appreciating the recent insights in more tumor-specific therapy. We here review the current standard of care for treatment of advanced and metastatic SS in adults and provide insights in the developments within the fields of targeted therapy and immunotherapy.

## Current pharmacological treatment options

### Chemotherapy

#### (Neo)adjuvant chemotherapy

The insights on (neo)adjuvant chemotherapy in STS have excellently been reviewed very recently [13, 14] and the most important trials are summarized in Table 1. In summary, in adults with localized STS of all localizations, chemotherapy in an adjuvant setting is not the standard of care, since many adjuvant STS trials, including SS, ultimately failed to prove overall survival benefit [20]. Neoadjuvant chemotherapy might be considered in specific situations, for example as induction therapy to enhance outcome of surgery in high-risk sarcoma of extremity and chest wall. Recent data suggest that also DFS may benefit from this approach. In this respect, two studies in SS are worth mentioning.

**Table 1:** (Neo)adjuvant chemotherapy

(Neo)adjuvant treatment	Population	Phase	Total *n* (SS)	Outcome	Year of publication
Histology-tailored neoadjuvant chemotherapy in 5 types of STS. For SS: high-dose ifosfamide (14 g/m^2^ in 14 days) every 28 days vs. epirubicine + ifosfamide 9 g/m^2^ in 3 days every 21 days [15••]	SS LMS mLPS MPNST UPS	III	287 (70)	DFS after 46 months histology-tailored chemotherapy 38% vs. standard group 46%, OS 89% vs. 64% (both significant, median follow-up 12.3 months, 70 events) Subgroup analysis: standard chemotherapy favorable for SS (HR 1.85, 95% CI 0.65–5.22, 17/70 events)	2017
Doxorubicin 75 mg/m^2^, ifosfamide 5 g/m^2^, and lenograstim q 3 weeks for 5 cycles adjuvant vs. active controle. 73% also had radiotherapy [16]	STS	III	351 (40)	Median OS 12.4 vs. 11.2 years (not significant) 5 years EFS: 52.9 vs. 54.9% 5 years OS 67.8 vs. 66.5%	2012
Doxorubicin 60 mg/m^2^ and ifosfamide 10 g/m^2^ for 3 neoadjuvant and 2 adjuvant courses [17]	STS	II	70 (20)	2 years PFS 75.7% 5 years PFS 63.8% 5 years OS 82.6%	2015
Doxorubicin 37.5 mg/m^2^ for 2 days and ifosfamide 3 g/m^2^ for 3 days followed by 2 cycles of ifosfamide 3 g/m^2^ for 2 days [18]	SS	II	138	3 years EFS 81.9% 5 years EFS 80.7% 3 years OS 97.2% 5 years OS 90.7%	2015
Not specified [19]	SS stage I–III	IV	544 pts. of whom 131 received chemotherapy	Hazard ratio chemotherapy for OS: - overall cohort: 0.95 (0.63–1.44) - stage I 4.80 (0.91–25.3) - stage IIA 1.32 (0.54–3.20) - stage IIB 0.36 (0.07–1.76) - stage III 0.56 (0.33–0.93), *p* = 0.028	2017

*SS* synovial sarcoma, *STS* soft tissue sarcoma, *MLPS* myxoid liposarcoma, *MPNST* malignant peripheral nerve sheath tumor, *UPS* undifferentiated pleiomorphic sarcoma, *DFS* disease free survival, *OS* overall survival, *PFS* progression free survival, *EFS* event free survival

A phase II trial exploring neoadjuvant treatment with doxorubicin 60 mg/m^2^ and ifosfamide 10 g/m^2^ for three neoadjuvant and two adjuvant courses in STS of the extremities, included 20 SS patients out of a total of 70 patients, and reported 2- and 5-year progression-free survival (PFS) rates of 75.7% (95% CI, 63.9–84.1%) and 63.8% (95% CI, 51.3–73.9%), respectively. The 5-year OS was 82.6% (95% CI, 71.3–89.7%). Protocol treatments were completed in 74% of the cases and toxicity was significant [17].

Results of a recent study in high-risk STS of extremity and chest wall, support the role of neoadjuvant combination chemotherapy, because of a gain in disease-free survival (DFS) [15••]. This study consisted of five cohorts of STS, with an SS cohort including 70 patients. Patients were randomized 1:1 to three cycles of standard treatment consisting of ifosfamide 3 g/m^2^ on days 1–3 and epirubicin 60 mg/m^2^ on days 1–2 of every 21 days vs. histology-tailored chemotherapy, which was in SS high-dose ifosfamide 1 g/m^2^ on days 1–14 of every 28 days [15••]. After a median follow-up of 12.3 months for the total study population (*n* = 287), the projected DFS at 46 months was 62% (95% CI 48–77) in the standard chemotherapy group and 38% [21••, 22, 23–26, 27•, 28•, 29, 30–37, 38•, 39, 40–44, 45••, 46, 47, 48, 49•, 50, 51, 52•, 53, 54] in the histotype-tailored chemotherapy group (stratified log-rank *p* = 0.004; hazard ratio 2.00, 95% CI 1.22–3.26; *p* = 0.006). Subgroup analysis indicated no preference for histology-tailored chemotherapy in the SS cohort (HR 1.85, 95% CI 0.69–5.22). Final OS data are awaited.

Another risk-adapted approach had been included in a study of the European Pediatric Soft tissue sarcoma Study Group (EpSSG). SS patients < age of 21 years were treated in the EpSSG NRSTS 2005 trial, which included 138 children and adolescents with SS [18]. Treatment was tailored according to risk group. Low-risk patients (R0 resection, tumor ≤ 5 cm in size) were treated with surgery alone, intermediate-risk patients (R0 resection and tumor > 5 cm in size, or R1 resection) with three to six courses of chemotherapy ± radiotherapy, and high-risk patients (R2 resection, no resection at all, or N1) were treated with six cycles of chemotherapy, surgery, and radiotherapy. Chemotherapy consisted of a maximum of four cycles of doxorubicin 37.5 mg/m^2^ for 2 days and ifosfamide 3 g/m^2^ for 3 days followed by two cycles of ifosfamide 3 g/m^2^ for 2 days. After a median follow-up of 52 months, the 3- and 5-year event-free survival (EFS) for all risk groups was 81.9 and 80.7%, and the 3- and 5-years OS was 97.2 and 90.7%, respectively. The only significant prognostic variable at univariate analysis was the risk group: 3-year EFS was 91.7% for low risk, 91.2% for intermediate risk, and 74.4% for high risk [18].

#### Palliative chemotherapy

##### First-line treatment

In STS, doxorubicin is still considered to be the standard first-line systemic therapy (Table 2), with response rates ranging between 16 and 27% and a median survival in clinical studies of nowadays approximately 18 months from start of first-line systemic treatment [62]. In the METAsarc study analyzing the outcome of 2225 STS patients in the real-life setting, the OS of the 150 SS pts. who started first-line treatment was 19.7 months [63, 64••]. In 2014, the EORTC STSBG 62012 study compared single-agent doxorubicin to the combination of doxorubicin and ifosfamide in STS showing a response rate of 13.6 vs. 26.5%, median progression-free survival (PFS) of 4.6 vs. 7.4 months and no significant difference in median OS (12.8 vs. 14.3 months) but significant more adverse events in the combination arm [21••]. In this trial, 64 out of 455 STS patients had SS (14%), but no histotype subgroup analysis was done.

**Table 2:** Palliative systemic therapy

Metastatic treatment	Population	Line of treatment	Phase	*n* (SS)	RR (%)	3 months PFS (%)	6 months PFS (%)	PFS (months)	OS (months)	Year of publication
Chemotherapy										
Doxorubicin vs. doxorubicin-ifosfamide [21••]	STS	1	III	454 (64)	14 vs. 26	–	–	4.6 vs. 7.4	12.8 vs. 14.3	2014
Doxorubicin-palifosfamide vs. doxorubicin[22]	STS	1	III	447 (22)	28.3 vs. 19.9			6.0 vs. 5.2	15.9 vs. 16.9	2016
Doxorubicin-evofosfamide vs. doxorubicin[23]	STS	1	III	640 (33)	28 vs. 18	–	–	6.3 vs. 6.0	18.4 vs. 19.0 SS: HR 0.32 (95% CI 0.14–0.73) *p* = 0·0043	2017*
Doxorubicin +/− olaratumab [45••]	STS	1	Ib-II	15 (Ib) + 133 (II). SS: 3	18 vs. 12	–	–	6.6 vs. 4.1	26.5 vs. 14.7 (HR 0.47 LMS, 0.56 other)	2016
Doxorubicin-dacarbazine +/− ifosfamide [55]	STS	1	III	340 (12)	17 vs. 32	–	–	4 vs. 6	12 vs. 13	1993
Doxorubicin vs. doxorubicin + trabectedin [24]	STS	1	II	113 (7)	17 vs. 17	–	–	5.5 vs. 5.7	13.7 vs. 13.3	2016
Doxorubicin vs. gemcitabine-docetaxel phase III [25]	STS	1	III	257 (11)	19 vs. 20	–	46.3 vs. 46.4	23.3 vs. 23.7 weeks	76.3 vs. 67.3 weeks	2017*
Ifosfamide [56]	STS	1, 2	II	114 (22)	All: 16 SS: 37	Median follow-up 3 years (actuarial estimate): 1 year PFS 15% 1 year OS 50% Median OS 55 weeks				2000
Trabectedin BSC [27•]	Translocation-associated STS	1–5	II	73 (18)	8 vs. 0%	70.3	44	5.6 vs. 0.9 months	Not reached vs. 8.0 (median FU 8.9)	2015
Eribulin [29]	4 cohorts STS including SS	2nd or 3rd	II	128 (19)	0	SS 21.1	–	–	–	2011
Gemcitabine-darcabazine vs. dacarbazine[32]	STS	≥ 2	II	109 (11)	12 vs. 4	56 vs. 37	–	4.2 vs. 2	16.8 vs. 8.2	2011
Targeted therapy										
Doxorubicin +/− olaratumab [45••]	STS	1	Ib-II	15 (Ib) + 133 (II). SS 3	18 vs. 12	–	–	6.6 vs. 4.1	26.5 vs. 14.7 (HR 0.47 LMS, 0.56 other)	2016
Cixutumumab (anti-IGF-1R antibody) [43]	5 cohorts STS including SS	All	II	113 (17)	–	SS 18	–	SS 6.4 weeks	–	2013
Cixutumumab (anti-IGF-1R antibody) [42]	Childhood cancer ≤ 31 years	All	II	114 (11)	All 4% SS 0%	–	–	–	–	2014
R1507 (IGF-1R antibody) [44]	STS	All	II	163 (23)	All 2.5% SS 0%	–	–	All 5.7 weeks SS 5.7	11 months	2014
Figitumumab (anti-IGF-1R antibody) [41]	Sarcoma	≥ 2	I	29 (5)	All 7% SS 0%	34%	28%	–	–	2010
Imatinib [57]	Sarcoma	All	II	158 (22)	SS 0%	–	–	SS 1.92	–	2009
Regorafenib vs. placebo [38•]	4 cohorts: SS, LPS, LMS and other	≥ 2	II	182 (27)	0	SS: 77 vs. 0%	SS: 38 vs. 0%	SS: 5.6 vs. 1.0	SS: no difference	2016
Pazopanib vs. placebo [35]	STS	2–5	III	372 (44)	6%	–	–	4.6 vs. 1.6	12.5 vs. 10.7	2012
Pazopanib [34]	4 cohorts STS	All	II	142 (38)	14%	SS 49%	–	161 days	–	2009
Sorafenib [40]	STS	All	II	145 (12)	–	–	–	3.2	14.3	2009
Gefitinib [58]	HER-1 positive SS	≥ 2	II	46	0	–	6%	6 weeks	–	2008
Panobinostat [59]	STS	≥ 2	II	48 (6)	0	20	13.2	1.7	10.3	2013
Immunotherapy										
Dendritic cell vaccinations [60]	Sarcoma	–	I–II	37 (2)	2.9	–	–	3 years PFS 2.9%	3 years OS 44.3%	2017
Autologous T cells transduced with an NY-ESO-1-reactive T cell receptor after lymphodepleting preparative chemotherapy [54]	NY-ESO-1(+) SS	≥ 2	Pilot	18	61	–	–	–	3 year OS: 38% 5 year OS: 14%	2015
Autologous T cells transduced with an NY-ESO-1-reactive T cell receptor after lymphodepleting preparative chemotherapy [53]	NY-ESO-1(+) SS	≥ 3	Pilot	6	67	–	–	–	–	2011
SYT-SSX-derived peptide vaccines + interferon-α every 14 days 6 times [61]	SS	–	–	21	0%	33%	–	–	–	2012

*SS* synovial sarcoma, *STS* soft tissue sarcoma, *OS* overall survival, *PFS* progression free survival, *PLS* liposarcoma, *LMS* leiomyosarcoma, *RR* response rate, *FU* Follow-Up, *HR* hazard ratio

As mentioned before, SS are considered to be more chemosensitive as compared to other STS histologies [7]. Sleijfer et al. reviewed ifosfamide in different EORTC studies and found an increased response rate of ifosfamide in SS compared to other histologies [11]. A recent EORTC review of 15 clinical STS trials investigated the outcome of chemotherapy in advanced SS patients, and included 313 SS patients out of 3330 STS patients. Nine out of these 15 trials investigated anthracyclines as monotherapy arm (*n* = 121 SS), 5 a combination of doxorubicin and ifosfamide arm (*n* = 112 SS), and 3 an ifosfamide monotherapy arm (*n* = 42 SS). The median PFS was significantly higher for SS patients compared to STS patients (6.3 vs. 3.7 months), as was the median OS (15.0 vs. 11.7 months) and the response rate (27.8 vs. 18.8%). Comparison of the response rates (combined complete (CR) and partial response (PR)) to the different chemotherapy schedules showed a response rate of 21.5% for anthracyclines alone, 32.2% for doxorubicin-ifosfamide, and 33% for ifosfamide alone. It was concluded that SS patients show superior responses to chemotherapy compared to STS patients and that, compared to anthracyclines alone, ifosfamide was (although not significant) the most active drug [9•].

Recently, two large phase III trials in STS patients testing new ifosfamide-related compounds, namely palifosfamide and evofosfamide, in combination with doxorubicin vs. doxorubicin alone, reported negative results [22, 23]. Both trials included 5% SS patients, but unfortunately reported no further details of this subgroup. Furthermore, a randomized phase II trial for doxorubicin plus trabectedin vs. doxorubicin alone in the first line was negative in 115 STS patients, of whom only 7 was with synovial sarcoma [24]. Very recently, a direct comparison between first-line doxorubicin and gemcitabine-docetaxel was made by Seddon et al. [25]. Five patients with synovial sarcoma out of 129 STS patients were treated with doxorubicin compared to 6 out of 128 patients treated with gemcitabine-docetaxel. After a median follow-up of 22 months, the median PFS of the total study populations was 23.3 vs. 23.7 weeks (HR 1.28, 95% CI 0.99–1.65, *p* = 0.06). The proportion of patients alive and progression-free at 24 weeks did not differ between those who received doxorubicin vs. those who received gemcitabine and docetaxel (46.3 vs. 46.4%). The number of synovial sarcoma patients in this study was too low to draw any significant conclusions.

In conclusion, in fit patients, data suggest that SS patients might benefit from first-line combination treatment of ifosfamide with doxorubicin over monotherapy doxorubicin, although—due to the rarity of the disease—this conclusion is not based on a prospective randomized study in SS.

##### Beyond first-line treatment

Several drugs are available for second-line and further treatment of advanced and metastatic STS (Table 2). The selection of a specific schedule is made on the basis of individual patient-based considerations, including age, performance score, histological subtype, tumor burden, pace of progression, main aim of treatment, toxicity experienced during first-line treatment, potential toxicity of the second-line treatment, etc. For SS, the second-line treatment of first choice for the majority of fit patients is ifosfamide, based on its known activity in SS. Alternative options are pazopanib and trabectedin in case patients are not fit enough for ifosfamide or have received the combination of doxorubicin and ifosfamide in first line. In some case, ifosfamide monotherapy can be considered after this first-line combination treatment, in particular when there has been a reasonable interval between the end of first- and start of second-line treatment. Single-center data on ifosfamide rechallenge in different STS subtypes showed the highest activity in SS [26]. The role of trabectedin in SS has been investigated in a Japanese randomized phase II study comparing trabectedin to best supportive care in translocation-related sarcomas, showed a significantly better response rate (8 vs. 0%), longer median PFS (5.6 vs. 0.9 months, HR 0.07), and longer median OS (not reached vs. 8 months, HR 0.42, median follow-up 8.9 months) for the total group [27•]. Eighteen out of the total of 73 patients had SS. This subgroup had a median PFS in favor of trabectedin (HR 0.14, 95% CI 0.03–0.68). A retrospective analysis of 61 SS patients treated with trabectedin in four European sarcoma reference centers reported a response rate of 15%, a median PFS of 3 months, and a 6-month PFR of 23% [28•]. Trabectedin in SS has never been tested in the context of a phase III trial [30]. Eribulin has also only been tested for SS in a phase II trial. This study included 128 patients in four cohorts, of which one involved the SS subtype, which included 19 patients. The primary endpoint was PFR at 12 weeks. The study was regarded positive for a cohort when the 12-week PFR was > 40% and negative < 20%. For SS, the 12-week PFR was 21.1%, for leiomyosarcoma 31.6%, and for adipocyte sarcoma 46.9% [29] The phase III trial subsequently only included leiomyosarcoma and adipocyte sarcoma [30]. Other chemotherapeutic options for STS in general include gemcitabine, dacarbazine, and cyclophosphamide-prednisone, without specific references for SS patients [31–33].

### Targeted therapy

In the past years, a lot of effort has been put into the design of a more effective and less toxic treatment modalities in SS patients. At present, however, pazopanib is still the only targeted drug approved for the treatment of STS, including SS, in the advanced setting after failure of standard cytotoxic therapy. Pazopanib is an oral, multi-targeted tyrosine kinase inhibitor directed against the receptor tyrosine kinases (RTKs) vascular endothelial growth factor (VEGFR) 1/2/3, platelet-derived growth factor (PDGFR) α/β, and KIT, thereby blocking tumor growth and inhibiting angiogenesis. In the first multistrata design with pazopanib in STS, the different PFRs at 3 months were 26% in adipocytic STS, 44%, in leiomyosarcomas, 49% in SS, and 39% in a stratum of other STS [34]. Based on this, the expectations for SS in the phase III study were high. The landmark PALETTE trial that led to the approval of pazopanib in STS in the advanced setting examined a total of 369 STS patients (12% SS). Patients were randomized 2:1 to receive either pazopanib 800 mg OD orally or placebo. Median PFS was significantly longer in the pazopanib-treated group; 4.6 vs. 1.6 months (HR 0.31, 95% CI 0.24–0.40, *p* < 0.0001), although no significant difference in median OS was detected (12.5 vs. 10.7 months, HR 0.85, 95% CI 0.67–1.11, *p* = 0.25). Partial remissions were observed in 6% of patients who received pazopanib, and another 67% had stable disease (SD) as best response [35]. For SS, the PFS and OS were promising when compared to other histologies, but due to the relatively low number of SS, no statistically significant differences were observed in this subtype. Pazopanib efficacy was also investigated in a retrospective analysis of the Named Patient Program called SPIRE study which included 24 SS patients out of 211 STS patients in total [36]. For the SS patients, the median duration on pazopanib in this study was 5.1 months and the median OS was 13.8 months. A phase I trial combining pazopanib with ifosfamide reported a PR in two patients with SS [37].

## Novel treatment options under development

### Novel targeted therapies in the clinic

In addition to pazopanib, various other (classes of) targeted therapy have been tested for efficacy in SS, including compounds directed against RTKs, epigenetic regulators, and immunomodulators. So far, the majority of preclinical and early promising results have failed to translate successfully to the clinic, with generally only short-lasting benefit in a small fraction of SS patients. However, some recent developments in the clinical and preclinical setting look more promising and will be further discussed.

#### (Receptor) tyrosine kinase targeted therapies

As the multi-RTK inhibitor pazopanib is at present, still, the only approved form of targeted therapy with evidence of clinical efficacy in SS, various studies examined the potential of targeting (other) RTKs or associated pathways in SS. To date, the highest clinical efficacy in SS has been observed for the multikinase VEGFR/PDGFR inhibitor regorafenib. The REGOSARC trial tested regorafenib 160 mg OD for 21 of each 28 days vs. placebo in four cohorts of doxorubicin pre-treated STS patients, including 1 SS cohort [38•]. In the SS cohort, PFS was 5.6 months (95% CI 1.4–11.6) with regorafenib vs. 1.0 (0.8–1.4) with placebo (HR 0.10 (95% CI 0.03–0.35) *p* < 0.0001) with 1 PR and 10 SDs out of 13 SS patients in the regorafenib arm. This effect was confirmed with a longer follow-up [39]. In a phase II trial with sorafenib, 12 SS out of 145 STS patients were treated with very limited efficacy [40]. Currently, a randomized EORTC phase II study ANITA comparing the oral angiogenesis inhibitor nintedanib with ifosfamide in second line is recruiting patients with advanced and metastatic STS, including SS (NCT02808247).

A phase III trial with an SS cohort (*n* = 95) comparing in a 2:1 ratio efficacy of the multikinase VEGFR/PDGFR inhibitor anlotinib to dacarbazine is currently recruiting patients (NCT03016819). Four phase II trials with an anti-IGF-1R-antibody did not show encouraging results in SS [41–44].

In the recent promising phase Ib-II study testing the combination of PDGFR antibody olaratumab plus doxorubicin vs. doxorubicin alone, only three SS patients were included [45••]. The phase III ANNOUNCE trial with similar treatment arms will hopefully have a reasonable subgroup of SS patients. Results of this study carried out in many STS subtypes, are awaited.

#### Epigenetic modifiers

The EZH2 inhibitor tazemetostat is currently the most studied epigenetic modulating drug in SS in the clinic. Tazemetostat activity is enhanced in integrase interactor 1 (INI1; aka SMARCB1)-negative tumors, in which INI1 loss allows EZH2 to become an oncogenic driver in tumor cells. INI1 deficiency specifically linked to the presence of the SS18-SSX1 fusion gene has been reported in SS in preclinical models [46, 47]. Accordingly, INI1 loss is a characteristic of SS tumor cell lines and tumors [48]. Preliminary data from ongoing adult phase I/II (NCT01897571) and phase II (NCT02601950) trials assessing single-agent tazemetostat activity in a variety of tumors including SS have confirmed clinical efficacy of tazemetostat in INI1-deficient tumors [49•, 50]. The three included SS patients in the phase I/II trial were, however, not INI1-negative and showed no response. As SS tumors are INI1-deficient rather than INI1-negative, cellular INI1 expression levels are reduced to varying degrees in SS, likely reflecting the variable tazemetostat treatment outcomes in SS [49•] The first results of a phase II trial with tazemetostat in SS and INI1-negative tumors have been presented at the annual meeting of ASCO 2017 (NCT02601950) [50]. In 33 treated SS patients with a median of two prior systemic treatments, best response was SD in 11 patients (33%) in which 5 patients lasted ≥ 16 weeks. No objective responses were observed. The protocol-defined success criterion at the end of study was not met. Additional studies examining tazemetostat efficacy in SS are currently ongoing. A phase II trial with vorinostat in 40 STS patients including three SS showed no objective responses, a median PFS of 3.2 months and median OS of 12.3 months [51].

#### Immunotherapy

In the field of immunotherapy, the use of engineered T cells directed against the NY-ESO-1 cancer/testis antigen, which is expressed in 80% of SS patients, has been the most promising approach in SS in clinical testing so far [52•]. Objective responses were reported in 4/6 heavily pre-treated, metastatic, NY-ESO-1-expressing SS patients treated with lymphodepleting preparative chemotherapy followed by autologous T cells transduced with an NY-ESO-1-reactive T cell receptor, including tumor regressions. Long-term follow-up of these patients confirmed objective responses in 11/18 SS patients (61%), including long-lasting partial and complete responses [53, 54]. A subsequent multi-cohort study with an updated protocol investigated effects of genetically engineered NY-ESO-1c259T cells recognizing an NY-ESO-1 derived peptide complexed with HLA-A2 in advanced, NY-ESO-1-positive SS patients. So far, promising efficacy and acceptable safety data have been demonstrated, including 1 CR and 5 PRs in the 12 treated SS patients of the first study cohort [65]. Interestingly, further data presented by D’Angelo at the annual meeting of CTOS 2017 reported responses in all four study cohorts, and the addition of fludarabine appeared to be required for optimal therapy response. Of note, in early 2016, the FDA granted breakthrough therapy designation for the affinity enhanced T cell therapy targeting NY-ESO for SS in the advanced setting and expressing NY-ESO-1 and HLA-A2 alleles. A variety of other clinical studies employing NY-ESO-1 in SS are currently under way (e.g., NCT02457650, NCT01343043).

The results of the Alliance A091401 study with the the PD1-inhibitor nivolumab and the combination of nivolumab and the CTLA4-inhibitor ipilimumab tested in 85 STS patients, included 4 SS patients. Objective responses were reported in 5% of patients treated with single-agent nivolumab and in 16% of the patients treated with the combination. No response was seen in SS. Median PFS and OS was 2.6 and 8.7 months for the single-agent nivolumab cohort and 4.5 and 11.2 months for the combination cohort (NCT02500797) [66••]. A second trial presented at ASCO 2017 combined low oral doses of cyclophosphamide with anti-PD1 pembrolizumab in different types of STS and GIST. In 50 evaluable patients, only three PRs were observed and very limited progression-free survival rates (NCT02406781) [67]. In the SARC028 study, pembrolizumab was administered in 85 patients with bone and soft tissue sarcoma, including 10 SS patients. For STS, median follow-up was 14.5 months. The objective response rate in the overall STS cohort was 18% and the 12-week PFR 55%. One out of 10 SS patients had a PR [68••]. An ongoing phase I–II trial combining pembrolizumab with doxorubicin in advanced or metastastic STS is currently recruiting patients (NCT02888665), as is a phase II study of Talimogene Laherparepvec (T-VEC) combined with pembrolizumab (NCT03069378). In the neoadjuvant setting, anti-PD-L1 (Durvalumab/MEDI4736) plus anti-CTLA-4 (Tremelimumab) and radiation is tested for high-risk STS (NCT03116529). The combination of RTK inhibitor axitinib and pembrolizumab is currently tested in specific types of STS including SS, after failure of anthracyclines (NCT02636725). As a first-line therapy, the combination of trabectedin, nivolumab, and anti-CTLA-4 ipilimumab is under investigation in a dose-finding phase I–II study for advanced STS (NCT03138161). A phase I trial in STS including SS with decitabine and autologous cancer testis antigen-specific dendritic cell vaccine has recently completed accrual (NCT01241162).

In conclusion, although it is too early to conclude on the efficacy of immunotherapy in SS, therapy based on engineered T cells showed exciting early results. The SARC028 trial reported one PR in SS [68••].

### Promising preclinical targeted therapies

Although as mentioned above, various preclinical studies have failed to translate into a significant benefit for SS patients, there are some remarks to make concerning both the set-up of these studies, as well as on the types of therapies included. Most preclinical studies performed in SS to date that failed to show a benefit in the clinic, were focused on drugs specifically aimed at (single) RTKs. This includes research on for example the targeting of the RTK IGF-1R with monoclonal antibodies, in which preclinical promising data did not translate into encouraging results in the subsequently executed four phase II clinical trials [41–44]. On reflection, this is not necessarily an unexpected result, as it has since been shown that SS only rarely depend on a single RTK for growth and harbor little to no recurrent mutations that could be associated with a driver signature [69•, 70•]. However, when combined with biomarkers capable of adequately identifying driver (RTK) signatures, implementation of such treatments could potentially translate into a clinical benefit in preselected subgroups of patients with a positive biomarker. The potential and proof-of-concept of such an approach was published recently, in which a global phosphoproteomics approach was used to identify activated targets and pathways in sarcoma. This approach identified the RTKs ALK and MET to be the drivers of 2/4 included SS cell lines, irrespective of the SS18-SSX driver, and pharmacological inhibition of these targets with clinically relevant inhibitors—such as crizotinib—dramatically affected SS tumor growth in vitro, and even led to complete tumor regressions in vivo. As in clinical SS specimens, these key driver signatures were also detected, albeit only in a small subset of patients, these (types of) therapies are likely only beneficial to a small subset of SS patients [69•]. In this light, other lines of (preclinical) research have aimed at ways to either directly or indirectly target the known driver characterizing SS, the SS18-SSX fusions. As SS18-SSX fusion genes have been shown to affect gene expression in an epigenetic fashion, the use of epigenetic-modifying compounds have been investigated in SS [71, 72]. Preclinically, the HDAC inhibitors vorinostat and quisinostat, and the EZH2 inhibitor tazemetostat were shown to specifically target SS in an SS18-SSX-dependent fashion [73–75]. Indeed, in the clinic, tazemetostat did show efficacy in SS patients as pointed out in the sections above, supporting the rationale to further investigate (combined) treatment regimens with tazemetostat, but also underlining the need to investigate the clinical potential of (other) epigenetic modifiers in SS. As mentioned before, the phase II study of vorinostat in refractory STS did not report an objective response in the three included SS patients [51], but a combination with for example the proteasome inhibitor bortezomib showed synergistic efficacy in SS models in vitro and in vivo and might be interesting [73]. Also, very recently, a synthetic lethal interaction was reported between the presence of the SS18-SSX fusion oncogene and sensitivity to inhibitors directed against the DNA damage response (DDR) kinase, ATR [76••]. Synergistic anti-tumor effects were further observed when combining the clinically relevant ATR inhibitor VX970 with PARP inhibitors or cisplatin, of which the latter combination was further examined in vivo and indeed enhanced SS tumor growth inhibition. As it was suggested that the presence of SS18-SSX fusions might introduce a vulnerability of these tumors to DDR inhibitors in general by creating a replication fork stress phenotype, and various DDR inhibitors are currently in clinical testing for other malignancies with encouraging results in certain histologies, this represents an interesting line of investigation that warrants further preclinical and clinical examination for SS [77]. In line with this hypothesis, specific sensitivity of SS cell lines to a variety of PARP inhibitors, including the clinically relevant compounds olaparib, talazoparib. and rucaparib, has been reported [76••, 78].

## Conclusion

Synovial sarcoma is an intriguing disease, which is relatively chemosensitive to standard first-line treatment and pazopanib and in a subset possibly also to other RTK inhibitors. It is still early days for epigenetic modulators, and immunotherapy, but successes are observed. Based on preclinical research, DDR inhibitors should also be tested in clinical studies.
